# Editors' Reply

**DOI:** 10.1371/journal.pmed.0020225

**Published:** 2005-07-26

**Authors:** Virginia Barbour, Barbara Cohen, Gavin Yamey

Steven Senn [1] raises several very important points about the publication of trials. In an attempt to improve transparency of reporting, we require authors to submit their protocol along with the trial so that it is available for reviewers and editors to compare with the journal article, and we encourage the protocol to be published with the trial so that readers can check these results for themselves. One problem in trial reporting is the relatively unstructured nature of trial reports in medical journals compared with, for example, Trial Bank (http://rctbank.ucsf.edu/). We are currently considering how we report trials at PLoS; a much more structured and, hence, more transparent report may make it much harder to hide results (or the lack of them).
